# Prevalence of thermophilic *Campylobacter* species in Swedish dogs and characterization of *C. jejuni* isolates

**DOI:** 10.1186/s13028-015-0108-0

**Published:** 2015-04-01

**Authors:** Mia Holmberg, Thomas Rosendal, Eva O Engvall, Anna Ohlson, Ann Lindberg

**Affiliations:** Department of Epidemiology and Disease Control, National Veterinary Institute, SVA, SE-751 89 Uppsala, Sweden; Department of Bacteriology, National Veterinary Institute, SVA, SE-751 89 Uppsala, Sweden; Växa Sverige, Box 30204, SE-104 25 Stockholm, Sweden

**Keywords:** Dog, *Campylobacter*, *C. upsaliensis*, *C. jejuni*, MLST, Prevalence

## Abstract

**Background:**

The aims of this study were to investigate the prevalence of *Campylobacter* species in Swedish dogs, to identify the species of the *Campylobacter* isolates and to genotype the *C. jejuni* isolates. Young and healthy dogs were targeted and the sampling was performed at 11 veterinary clinics throughout Sweden from October 2011 to October 2012. Faecal swab samples were collected and sent to the laboratory at the National Veterinary Institute (SVA) for isolation of *Campylobacter*, speciation and genotyping.

**Results:**

*Campylobacter* spp. were isolated from 67 of the 180 sampled dogs which yields an overall prevalence of 37%. The most prevalent species of *Campylobacter* among the participating dogs was *C. upsaliensis* with 52 of the 67 identified isolates. A lower prevalence was observed for *C. jejuni* with seven identified isolates and one isolate was identified as *C. helveticus*. Multi-locus sequence typing (MLST) was carried out on the seven *C. jejuni* isolates and all sequence types that were found are also commonly found in humans. The dogs were divided into three age groups; 1) under 12 months, 2) 12 to 23 months and 3) 24 months and older. The highest prevalence was found in the two younger age groups. Dogs shedding *C. jejuni* were between 3–12 months of age while dogs shedding *C. upsaliensis* were found in all ages.

**Conclusions:**

The present investigation finds that *Campylobacter* spp. known to cause campylobacteriosis in humans are present in Swedish dogs. The results suggest an age predisposition where dogs under 2 years of age are more likely to shed *Campylobacter* spp. than older dogs. The most commonly isolated species was *C. upsaliensis* followed by *C. jejuni,* which was only detected in dogs up to 12 months of age. All *C. jejuni* isolates identified in the present study were of the same MLST types that have previously been described both in humans and in animals. The awareness of the *Campylobacter* risk of healthy young dogs may be an important way to reduce the transmission from dogs to infants, young children and immunocompromised adults.

## Background

Campylobacteriosis is the most commonly reported zoonotic disease and the most common cause of bacterial enteritis in humans in many countries throughout the world [1]. In 2013, there were 8114 notified human cases of campylobacteriosis in Sweden. Of these, 41% were considered to be domestically acquired [2]. Humans can be infected with *Campylobacter* by several routes and the bacteria are commonly found in a wide range of animals including cats and dogs. Especially in young dogs (<1 year), *Campylobacter* is often found in faecal samples and the dogs usually shed the bacteria without showing any clinical signs [3,4]. Several studies have reported the presence of *Campylobacter* spp. in both healthy dogs and dogs with diarrhoea, but *Campylobacter* is generally not considered to be pathogenic to dogs [5-8]. No association was found between presence of *Campylobacter* and diarrhoea in a Norwegian study on dogs [8] whereas a study in Canada found significant higher infection rate in diarrhoeic dogs compared with healthy dogs [5]. In an investigation of dogs in Ireland, diarrhoeic dogs were more likely to be *Campylobacter* positive than dogs without diarrhoea, but the dogs with diarrhoea also had concurrent gastrointestinal parasites, inflammatory bowel disease or diabetes [9]. A study of dogs in Barbados found no difference in clinical disease in dogs with and without presence of *Campylobacter* in the faeces, but indicated that co-infection with parvovirus and *Campylobacter* was common [10]. An association between occurrence of diarrhoea and *Campylobacter* infection was described in a previous Swedish investigation of dogs from 1979, and 63% of the dogs with diarrhoea also had antibodies to canine parvovirus [11]. The true role of *Campylobacter* in canine gastroenteritis is uncertain as the bacteria may be found in clinically healthy dogs or often as a co-infection or intestinal carriage in diseased dogs.

In most studies, the predominant *Campylobacter* species isolated from dogs is *C. upsaliensis* and dogs are regarded as an important reservoir for this species [3-6,9,12]. The second most common *Campylobacter* species isolated from dogs, in many populations, is *C. jejuni* [3,4,6,9,12], which is also responsible for the majority of human infections [13,14]. The reported prevalence of *Campylobacter* in dogs varies widely between studies, ranging from 22% to 100% and is reported to depend on factors such as the age, diet and housing of the dogs. Previous prevalence investigations of *Campylobacter* in dogs have also varied by study design and method of bacterial isolation.

Human campylobacteriosis is frequently attributed to contact with contaminated food (especially poultry meat) or water [15,16]. In several studies, direct contact with pet animals has also been identified as a possible source of human *Campylobacter* infection [15,17-19]. Presence of a puppy in the household has been identified as risk factor for campylobacteriosis, especially in young children [20,21]. In a study by Wolfs *et al.* [19] evidence was presented for transmission of *C. jejuni* from a dog to a 3-week old infant. However, a study by Studahl and Andersson [16] did not find a significant association between human campylobacteriosis and contact with dogs.

The present survey is part of an ongoing more comprehensive *Campylobacter* source attribution study in Sweden. Strains from human cases of campylobacteriosis were collected during the same time period as samples were taken from dogs, cattle, pigs, sheep, poultry and wild birds. Other relevant sources of human campylobacteriosis such as retail poultry meat, raw water and bathing water were also sampled during this period.

The aim of this study was to update our knowledge on the prevalence of *Campylobacter* spp. in young dogs in Sweden by collecting samples from healthy, young dogs throughout a year. The aim was also to identify the species of the *Campylobacter* isolates and to genotype the *C. jejuni* isolates by multi-locus sequence typing (MLST) to enable comparison between *C. jejuni* isolates from dogs and humans.

## Methods

### Study population and sampling

#### Selection of clinics to participate in the study

The sampling of dogs was performed at veterinary clinics throughout Sweden from October 2011 through October 2012. The geographical regions (counties) to be included in the survey were selected depending on the number of reported human cases of campylobacteriosis in recent years (based on data from the Public Health Agency of Sweden, www.folkhalsomyndigheten.se). The counties with the highest incidence of human campylobacteriosis were prioritised but the aim was also to cover a large part of the country. Requests for participation in the sampling process were sent to 53 veterinary clinics that had a previously established contact with the laboratory at Department of Bacteriology at the National Veterinary Institute (SVA, www.sva.se). A selection of 11 was made from the 18 veterinary clinics that had agreed upon participation in the study. One of them, located in the north of Sweden was included in the survey despite the lower incidence of human campylobacteriosis in favour of the better geographic coverage.

#### Sampling procedure

The requirements for dogs to be sampled were that they were under the age of two, weaned and healthy with no signs of diarrhoea. Young dogs were targeted because the prevalence of *Campylobacter* spp. is likely to be highest in young animals [4,6,22]. The aim was to collect a total of 200 samples in the study in order to obtain approximately 100 isolates. Based on a previous study in Sweden by Engvall *et al.* [4] an overall isolation rate of *Campylobacter* spp. of around 50% was expected. The clinics were instructed to sample one to two dogs per month. Each dog was sampled only once during the sampling period. Faecal samples were collected from freshly voided faeces and sent to SVA on swabs in Amies transport medium with charcoal (Amies agar gel swabs – with charcoal, Copan, Italy). The swab samples were sent to SVA by ordinary mail at the day of sampling for isolation of *Campylobacter*, species identification and genetic subtyping of *C. jejuni* isolates. Written consent of the animal owners was obtained for sampling along with details about the age of the dog and postal code of the owner.

### Isolation and species identification of *Campylobacter*

Swab samples were cultured on modified charcoal, cefoperazone, desoxycholate agar (mCCDA), (Oxoid Inc, Basingstoke, Hampshire, UK) and incubated at 41. 5 ± 1.0°C for up to 5 days in a microaerobic atmosphere created by use of Campygen (Oxoid Inc) or Anoxomat (Advanced Instruments, Inc., Norwood, Massachusetts, USA). Preliminary identification of *Campylobacter* spp. was based on colony and microscopic morphology and the following phenotypic and biochemical characteristics and tests; motility, oxidase-, catalase-, hippurate and indoxyl acetate reactions. Strains confirmed as *Campylobacter* spp. were stored at −80°C until further identification. Identification of *C. jejuni* was mainly based on a positive hippurate test. The species identification of all hippurate-negative isolates was done by mass spectrometry, time of flight, Maldi-Tof [23]. All isolates that were identified as *C. upsaliensis* by Maldi-Tof were further tested by polymerase chain reaction (PCR) for confirmation [24,25].

### Genotyping of *C. jejuni*

MLST was carried out as previously described by Dingle *et al.* [26] on the isolates identified as *C. jejuni*. Alleles, sequence type (ST) and clonal complex were assigned using the pubMLST database (http://pubmlst.org/campylobacter). Sequence types that shared four or more alleles were considered to belong to the same clonal complex. Because the present study is part of a larger comparative study of *C. jejuni* between various sources in Sweden, genotyping was restricted to this *Campylobacter* species.

## Results

**Figure 1:**
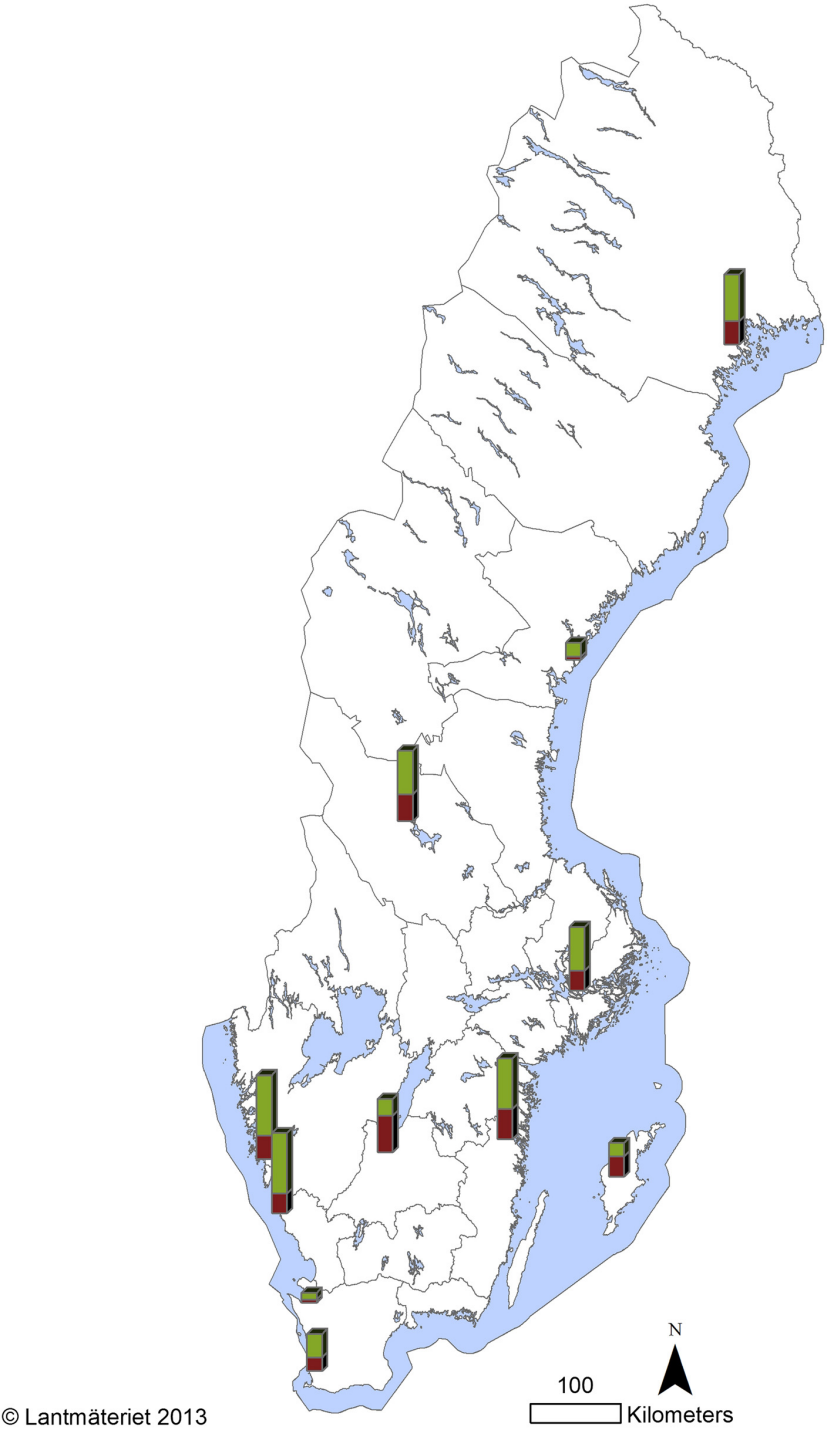
**Spatial distribution of veterinary clinics where dogs were sampled.** The height of the bars represents the number of sampled dogs (red = positive samples, green = negative samples).

**Figure 2:**
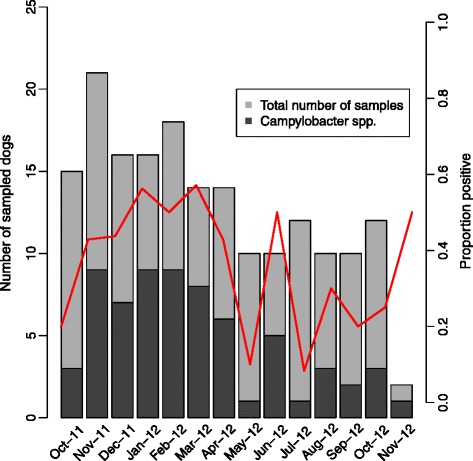
**Total number of samples and samples with** ***Campylobacter*** **species per month.** Proportion positive samples is indicated by the red line.

In total, 180 dogs were sampled from the 11 participating veterinary clinics. The sampling period was from October 2011 to October 2012; however, two samples received in November 2012 were also included in the study. For practical reasons, the number of samples received per month varied between the participating clinics. One clinic only sent in samples from one month. The location of the clinics and number of sampled and positive dogs per clinic is illustrated in Figure 1. One of the positive samples lacked information about clinic on the referral form. Most samples were received during the first seven months of the sampling period (October – April). The highest proportion of positive samples occurred in the winter months with a peak in March 2012 (57%). The number of samples per month and positive samples per month are shown in Figure 2.

**Figure 3:**
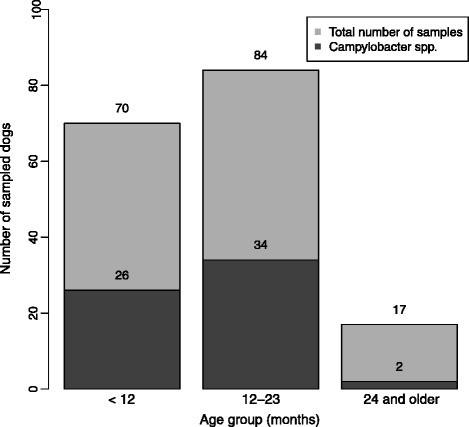
**Number of dogs in different age groups that tested positive or negative for** ***Campylobacter*** **species.**

The age distribution of the sampled dogs ranged from one month to 11 years. The aim was to sample dogs under the age of two, although 17 samples turned out to be from dogs between 2 and 11 years and 9 samples lacked information about age. The average and median age was 12 months. The dogs were divided into three age groups; 1) under 12 months, 2) 12 to 23 months and 3) 24 months and older. Number of samples and prevalence of *Campylobacter* in the different age groups are shown in Figure 3. Highest prevalence was found in the two younger age groups (37% and 40% for group 1 and 2, respectively) and the lowest in age group 3 (12%).

*Campylobacter* spp. were isolated from 67 of the 180 sampled dogs which yields an overall prevalence of 37% in this material. The most common species of *Campylobacter* isolated from the dogs was *C. upsaliensis* with 52 of the 67 identified isolates (78%). A lower occurrence of *C. jejuni* was observed, with 7 identified isolates (9%) and 1 isolate (1%) was identified as *C. helveticus.* All *C. jejuni* samples were found in dogs up to 12 months of age. In addition there were 7 isolates of *Campylobacter* species that did not survive the storage process, prior to the speciation. These isolates were all hippurate negative which indicates that they were most likely not *C. jejuni*, but could not be identified to species level by Maldi-Tof or PCR.

Results from the MLST are shown in Table 1. The dogs shedding *C. jejuni* were sampled at different locations except for two that were sampled in the same clinic. All seven isolates had different ST types. However, two dogs, sampled at different locations, were shedding *C. jejuni* of the same clonal complex, ST Clonal complex 21.

**Table 1:** **Results from multi-locus sequence typing (MLST) of** ***Campylobacter*** **isolates from dogs**

**Dog**	**Age (months)**	**Location of veterinary clinic**	**County of Sweden**	**MLST**	**MLST-complex**
1	12	Västra Frölunda	Västra Götaland	50	ST-21 complex
2	4	Jönköping	Jönköping	883	ST-21 complex
3	12	Jönköping	Jönköping	52	ST-52 complex
4	4	Ängelholm	Skåne	45	ST-45 complex
5	3	Varberg	Halland	122	ST-206 complex
6	12	Gamleby	Kalmar	257	ST-257 complex
7	4	Gammelstad	Norrbotten	677	ST-677 complex

## Discussion

In this study, 37% of the tested dogs were found to be positive for *Campylobacter.* This proportion is similar to the previous prevalence estimates of other studies [6,9,27]. However, as we targeted young dogs and the mean age of the sampled dogs was 12 months this estimated prevalence may not be representative for the entire Swedish dog population. Moreover, the study design was such that each dog was only sampled once and only one isolate per dog was analysed. In longitudinal studies with another type of study design where dogs were sampled at several occasions considerably higher prevalences of 73-100% have been reported [3,28]. The most common *Campylobacter* species among the tested dogs (52/180 dogs) was by far *C. upsaliensis* which is in agreement with many previous studies on dogs where relatively high prevalences of *C. upsaliensis* have been found in many different populations and countries [5,7,9,22,28]. The lower prevalence (4%) that was observed for *C. jejuni* (7/180 dogs) is similar to some studies [12,27,29,30], but lower than in a previous Swedish study by Engvall *et al.* [4] where 11% of the dogs were positive for *C. jejuni*. However, in the previous study all samples were cultured on three selective plates (two CAT and one Preston agar plate). This probably contributed to the higher isolation rate compared to the present study. A recent study in Switzerland by Amar *et al.* [30] found a low prevalence of *C. jejuni* (6.3%) and an even lower prevalence (5.9%) of *C. upsaliensis* in healthy dogs. A higher prevalence of *C. jejuni* has been reported in stray dogs [10,28], which may be due to a different exposure to environmental sources of *Campylobacter* than what household dogs are normally exposed to. In a study from Spain, 19% (20/105) of dogs under 2 years of age living in a household, were found to have *C. jejuni* [22]. In the present study, one sample was identified as *C. helveticus* which is not so often identified in samples from dogs, but has previously been associated with cats [29].

Although *C. upsaliensis* is not isolated very frequently from humans in routine investigations it is possible that available data underestimate the prevalence due to the methods that are used at the public health laboratories which are primarily developed to detect *C. jejuni* and *C. coli.* In Sweden and many countries in Europe, human clinical *Campylobacter* isolates are not identified to species level which might contribute to a lack of data regarding the prevalence of *C. upsaliensis* in humans [31]. The role of *C. upsaliensis* in human disease is not very well established but it has been shown that *C. upsaliensis* can be a cause of gastroenteritis in both adults and children [32,33]. Labarca *et al.* [34] found that *C. upsaliensis* was the second most frequently isolated species in humans after *C. jejuni.* The authors also found that three dogs living in the households of two human patients infected with *C. upsaliensis* had the same *Campylobacter* species isolated in their stool specimens, but were not from the same clonal complex [34]. A study in Belgium reported that an outbreak in four day care centres was caused by *C. upsaliensis* [35]*.* Damborg *et al.* [36] found that a cluster of human *C. upsaliensis* strains was unrelated to dog strains of *C. upsaliensis* examined in the study by AFLP fingerprinting. However, the human and dog samples were not collected in the same countries which makes it difficult to interpret the results with regard to host specificity.

The age of each dog at sample collection was recorded and the dogs were divided into three age categories for comparison of prevalences. The results were in agreement with many other studies that have reported higher prevalence of *Campylobacter* in younger dogs or puppies compared with adult dogs [6,9,12,22,27,37]. These results suggest an age predisposition where young dogs are more susceptible to colonisation, possibly due to the development of immunity with age. Senior dogs have also been found to be at risk for *Campylobacter* colonisation [27,28]. Wieland *et al.* [29] found a significant association with age and *C. upsaliensis* but no association between age and presence of *C. jejuni*. Dogs shedding *C. jejuni* in the present study were all young, between 3–12 months while dogs shedding *C. upsaliensis* were found in all age categories, which is similar to what was described by Hald *et al.* [3].

Seasonality that is observed in human campylobacteriosis with peaks during the summer months [31] was not observed in this material as the highest incidence was observed in the winter months with a peak in March. Due to the low number of samples per month it is not possible to draw any conclusions regarding seasonality based on our findings. Carbonero *et al.* [22] found a significantly higher prevalence of *C. jejuni* in dogs during spring compared to winter. They also reported a higher prevalence of *C. upsaliensis* during the summer. Hald *et al.* [3] did not find seasonal variation in carrier rates among dogs in a longitudinal study.

Molecular typing techniques enable comparison of sequence types (STs) between humans and the potential source of *Campylobacter*. In our study, seven *C. jejuni* samples were isolated and further subtyped by MLST. The results show high heterogeneity as all isolates were of different STs and only two isolates (from dogs sampled in different areas) belonged to the same clonal complex, ST-21. Since only 7 *C. jejuni* isolates were identified and typed, the true extent of the *C. jejuni* population diversity in Swedish dogs cannot be estimated from this material. Mughini Gras *et al.* [21] showed a high degree of overlap between human and pet (dog and cat) *C. jejuni* STs. They identified two cases where identical *C. jejuni* STs (ST45 and ST658) were isolated from dogs and their owners. Four of the STs identified in the present study (ST50, ST45, ST257 and ST122) belong to those more frequently found STs in pet owners in the study by Mughini Gras *et al.* [21]. Manning *et al.* [38] found that a majority of the identified *C. jejuni* ST-complexes overlap between human and various animal sources. When comparing to the *Campylobacter* MLST database of *C. jejuni* isolates (http://pubmlst.org/campylobacter) all seven STs that were found are common human STs. In the study by Amar *et al.* [30] the most frequent STs identified in dogs include ST45 and ST21 that was found also in the present study. Parsons *et al.* [28] also reported that ST45 was the most common complex in dogs at a rescue kennel. Many studies have identified ownership or close contact with dogs as a potential risk factor for human campylobacteriosis. However, despite the use of molecular information it is often difficult to know whether the bacteria is transferred from dog to human or human to dog or was acquired from the same common source.

In general, it is likely that dogs and humans are exposed to common sources of *C. jejuni.* The relatively low prevalence of *C. jejuni* in dogs in this study suggests that the importance of dogs for human infections compared with other sources such as food products may be low. However, dogs and in particular puppies are likely to have close contact with their owner and children in a family. Considering this it is important to note that all *C. jejuni* positive dogs were puppies or young dogs up to 12 months old. As the prevalence of *C. upsaliensis* in dogs under two years of age is relatively high there is a risk for transmission of zoonotic *Campylobacter* from dogs to humans and especially young children that are more susceptible for infection.

## Conclusion

The present investigation finds that *Campylobacter* spp. known to cause campylobacteriosis in humans are present in Swedish dogs. The results suggest an age predisposition where dogs under 2 years of age are more likely to shed *Campylobacter* spp. than older dogs. The most commonly isolated species was *C. upsaliensis* followed by *C. jejuni,* which was only detected in dogs up to 12 months of age. All *C. jejuni* isolates identified in the present study were the same MLST types as had been previously described both in humans and in animals. Further investigation is necessary to determine the similarity between these dog *C. jejuni* MLST types and those found in humans in Sweden during the same period. The awareness of the *Campylobacter* risk of healthy young dogs may be an important way to reduce the transmission from dogs to infants, young children and immunocompromised adults.
